# Unlocking stress and forecasting its consequences with digital technology

**DOI:** 10.1038/s41746-019-0151-8

**Published:** 2019-07-31

**Authors:** Sarah M. Goodday, Stephen Friend

**Affiliations:** 1 4YouandMe, Seattle, WA USA; 2 Department of Psychiatry, University of Oxford, Oxford, UK

**Keywords:** Predictive markers, Biological models

## Abstract

Chronic stress is a major underlying origin of the top leading causes of death, globally. Yet, the mechanistic explanation of the association between stress and disease is poorly understood. This stems from the inability to adequately measure stress in its naturally occurring state and the extreme heterogeneity by inter and intraindividual characteristics. The growth and availability of digital technologies involving wearable devices and mobile phone apps afford the opportunity to dramatically improve measurement of the biological stress response in real time. In parallel, the advancement and capabilities of artificial intelligence (AI) and machine learning could discern heterogeneous, multidimensional information from individual signs of stress, and possibly inform how these signs forecast the downstream consequences of stress in the form of end-organ damage. The marriage of these tools could dramatically enhance the field of stress research contributing to impactful and empowering interventions for individuals bridging knowledge to practice, and intervention to real-world use. Here we discuss this potential, anticipated challenges, and emerging opportunities.

## Introduction

The significant contribution of stress to the development of disease is unequivocal. Often labeled as the silent or proxy killer, chronic stress is a major underlying origin of the top leading causes of death, globally.^1^ The magnitude and ways that stress affects individuals and populations is far reaching with impacts that can be seen as early as during the embryonic stage spanning into adulthood.^2^ Despite the concerted effort from cross-disciplinary research to understand the stress-related underpinnings of disease, explanatory mechanisms to inform individualized intervention targets remain an enigma. While it is clear a link between a wide range of psychological to objective biological measures of stress and chronic disease exists, the mechanistic explanation of how stress over time leads to end-organ damage is unclear. This stems from several roadblocks rooted in: (1) ambiguous definitions of stress; (2) the inability to adequately measure stress in its naturally occurring state; (3) the inability to discern measures of the stress response given the extreme heterogeneity by inter and intraindividual characteristics; and (4) lack of knowledge on the interplay between subjective and objective measures of stress. Further, the emphasis on aggregate versus individual level information, and binning of symptoms into rigid diagnostic categories has undoubtedly hampered progress in understanding individual trajectories towards end-organ damage.

The growth and availability of digital technologies involving wearable devices and mobile phone apps afford the opportunity to dramatically improve measurement of the biological stress response in real time. In parallel, the advancement and capabilities of artificial intelligence (AI) and machine learning could discern heterogeneous, multidimensional information from individual signs of stress, and possibly inform how these signs forecast the downstream consequences of stress. The marriage of these tools could dramatically enhance the field of stress research contributing to impactful and empowering interventions for individuals-bridging knowledge to practice, and intervention to real-world use.

## Defining stress

There is no single agreed upon definition of stress, although the term tends to be associated with emotional, mental, and physiological strain in response to a real or perceived demand or threat. Linking causes of stress to disease has been challenging given the dramatically different effects on individual biological stress responses and the many different ways that individuals cope with stress resulting from genetics, personality, and environment.^3^ Further, it is unclear whether perceived subjective experiences (e.g., feeling stressed) of stress map on to the body’s biological short- or long-term reaction to stress that infers risk of disease. Hans Selye’s original definition of stress (and among one of the first conceptualizations of stress in relation to health) was rooted in the biological reaction to a stressful exposure arguing that “stress is not what happens to you, but how you react to it”.^4^ While it is appreciated that there is no single “correct” definition for stress, a deserving question is what component of the stress process (e.g., perceiving as feeling stressed vs. the body’s reaction to stress, or combinations of both) is important in terms of disease-modifying pathways and where along the pathway should we target interventions and tools to maximize reduction of the deleterious effects of stress?

The needed data to inform such a question would be continuous longitudinal information from subjective experiences of stress, mapped on to continuous objective measures of stress, and spanning enough time for consequences of stress to accrue, which could be months to years for some conditions. Simply put, without the aid of remote capture digital monitoring we cannot achieve this scale of data collection. In fact, most attributes of the human stress response cannot be accurately captured through traditional methodologies. The biological stress response is analogous to an orchestrated symphony^5^—complex and involving several interconnected players including neuroendocrine, cardiovascular, metabolic, and immune systems that adaptively follow a harmony in response to demands or threats under healthy brain conditions. Conscious and subconscious momentary responses to stress repeated over time can cause dysregulation in normal adaptive hormonal and physiological responses to stress contributing to risky symptoms (high blood pressure, inflammation depression, cognitive decline, and sleep problems) called by some as allostatic load,^6,7^ which in turn contribute to downstream consequences (chronic disease). It is often presumed that the acute stress response spans milliseconds to seconds, however, such exposures can produce delayed molecular changes days after the initial stress exposure,^8^ further compounding the need for continuous longitudinal information of these biological responses. These processes culminate over time, although may also accelerate during high-stress activation periods throughout the lifespan such as pregnancy^9^ reflecting useful periods to understand stress-related processes underlying disease. Individuals reactions to both subjective and objective stress are likely modified by internal (e.g., personality and epigenetics) or external (social support and empathetic relationships) coping factors and vary with great complexity given these inter and intraindividual differences. Until recently, we have had little means to measure, and discern the complexity associated with individual stress responses, and how these responses forecast disease.

## The potential power of integrated digital tools

**Fig. 1:**
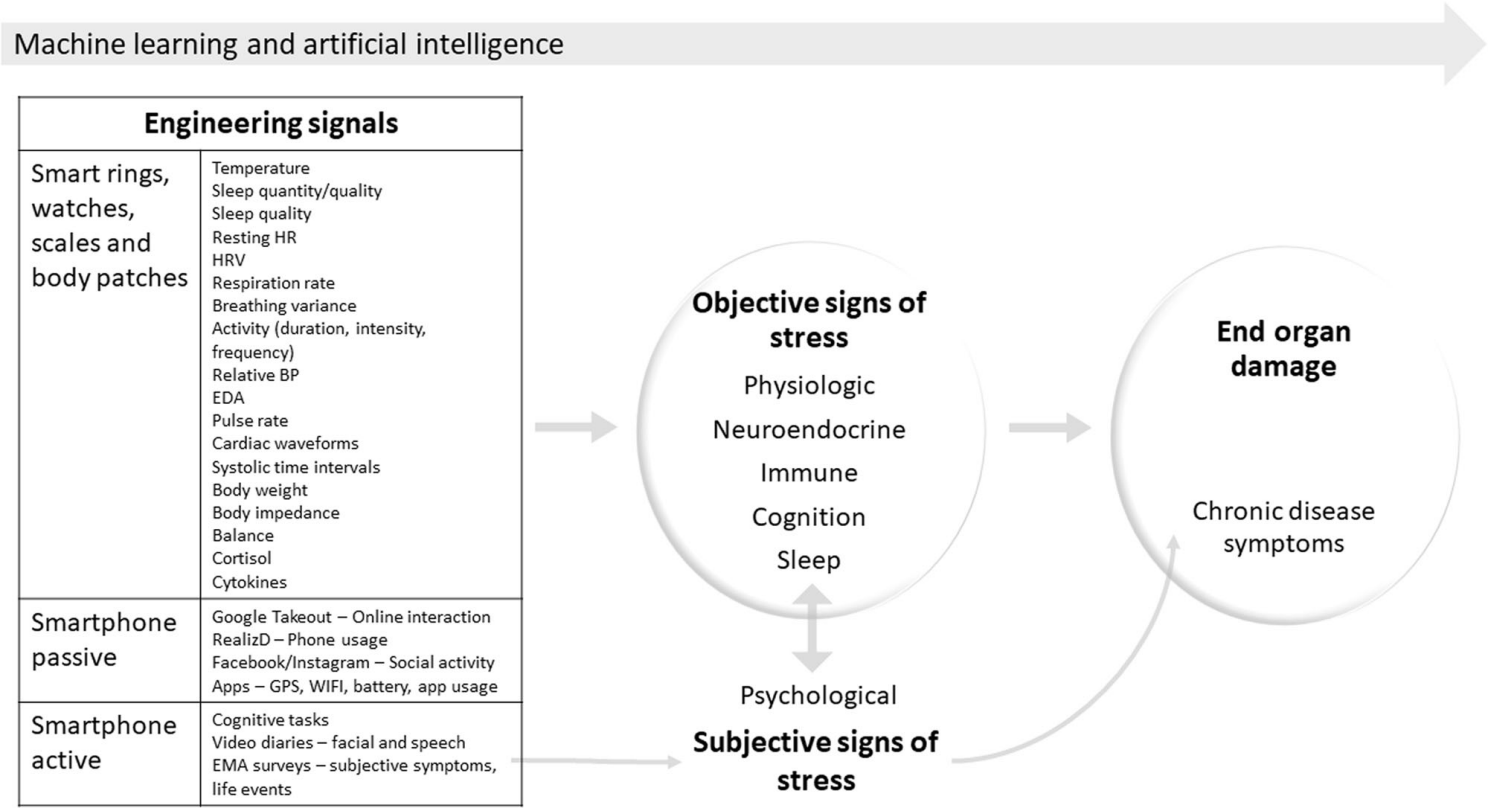
Engineering signals from wearable devices to signs of stress to end-organ damage

Digital data from connected wearable devices and smartphone apps offer an exciting avenue to explore the complex and dynamic biological stress response in its natural form (Fig. 1). It is projecting that by the year 2020, 50 billion individuals globally will own connected smartphones and will own over six connected devices.^10^ Smartwatches, rings, body scales, and vests have the capability of producing rich volumes of longitudinal information on physiological proxies of the autonomic nervous system stress response, such as resting heart rate, heart rate variability, body temperature, electrodermal activity, and relative blood pressure,^11–13^ while body patches that measure cortisol^14^ and inflammatory cytokines^15^ and eye-tracking glasses and wireless EEG caps are getting closer to measuring additional objectives measures of stress in real-world settings.^16^ Information from smartphone apps and sensors, both passive (phone use, social communication patterns, sleep, and location) and active (cognitive tasks) could reflect digital proxies of objective measures of stress.^13^ Realizing this potential, smartwatches that detect physiological signals of stress and inform users when these signals occur have recently been developed. These approaches to measuring the biological stress response make a giant leap in overcoming the feasibility and adherence limitations of past approaches traditionally confined to intrusive specimen collection at infrequent times at home or to aperiodic clinic visits.

There is a growing body of evidence supporting the potential capabilities of connected digital devices in detecting signs of stress via a wide array of digital biomarkers. For example, sensors with global-positioning systems are able to detect changes in movement patterns, activity, and time spent in different locations, while smartphone apps are able to collect passive data in the form of change in device or app usage and number of calls or texts among many other features. These digital biomarkers have been linked to perceived stress and changes in mood in university populations,^12,13,17^ several psychiatric populations^18–20^ and Parkinson’s disease.^21^ Audio recordings from smartphones are able to detect rate of speaking, changes in pitch, pauses, potentially reflecting vocal markers of stress^22^ and these features have been shown to be associated with objective measures of stress captured through smartwatches during stressful scenarios in real life.^23^ Further, through video data, smartphones have the capabilities of facial emotion recognition that are able to detect shifts in subjective reported mood states.^24^ Participation in online social networking may influence signs of stress and well-being. Proxies of social support have been suggested in the form of number of online interactions and views, “likes” etc. although these general forms of online interaction have not been found to be associated with health outcomes and some forms may pose harm, following theories of social comparison.^25^ However, interaction with individuals with strong ties has been linked to improvement in self-reported well-being.^26^

The collection of these digital objective measures of stress in parallel to smartphone apps that repeatedly collect subjective experiences of stress could offer an opportunity to delineate the concordance or discordance between feeling stressed and the body’s biological acute and long-term response to stress. This capability also opens doors to novel inquiries about new models of stress and disease centered on the potential importance of momentary feelings of control or uncertainty^27^ during exposure to stress and how this feeling maps on to objective stress and downstream deleterious consequences.

Innovative statistical approaches are needed to translate this knowledge in a way that is useful for both individuals and healthcare providers. Modern machine learning and AI techniques are powerful means to work with complex relationships (unknowns, unknowns as described in the Cynefin framework^28^) in high-dimensional data and learn from them.^29^ In turn, the hope is to shift towards complicated relationships, where cause and effect might be elucidated with the “expert” help of AI. The big data emanating from wearable devices and smartphone apps produces a challenge for machine learning and AI specialists to produce methodologies capable of estimating individual level trajectories of disease that are interpretable. While supervised methods such as recurrent neural networks are able to forecast future disease from prior history, the interpretability of these methods is sacrificed, leaving the mechanistic processes of disease progression unknown and findings relevant to a population level interpretation. Fortunately, machine learning and AI approaches are rapidly developing that attempt to address these methodological limitations capable of accommodating complex data structures involving high-frequency longitudinal individual level data with high inter and intra variation,^30–32^ and that could make individual predictions for impending disease.^34–36^ Discoveries from machine learning and AI in this context could lead to new insights of trajectories of disease that could meaningfully inform revisions to the current categorical nosology, lending insight into new endotypes defined as subtypes of disease reflecting the underlying pathophysiology rather than phenotypic observable characteristics, initially applied in the context of asthma.^33^

## Emerging opportunities

The number of wearable devices available on the market has exploded in recent years^10^ and several systematic reviews outline the promise of these technologies for a wide variety of chronic conditions, such as diabetes,^37^ parkinsons,^38^ and cancer.^39^ Although, many of these applications surround detection of end-organ symptoms to be used in clinic, or are used for chronic disease management, such as continuous glucose-monitoring sensors for diabetes management.^37^ The clinimetric properties of most wearable technologies is unknown as well as their capabilities for detecting early symptoms and stress that might help not only clinicians, but also individuals to monitor early warning signs for prevention.

Still, these complimentary approaches could benefit a wide variety of chronic health conditions targeting a diverse array of end-organ damage. The acute impact of stress may have proximal effects on chronic disease symptoms from weakened stress-mediating symptoms and by direct effects on key triggers of disease activity, such as brain lesions in multiple sclerosis (MS),^40^ and inflammatory markers in irritable bowel disorders.^41^ Promising findings from a randomized controlled trial of MS patients found that stress management therapy reduces gadolinium-enhancing brain lesions associated with MS flare ups.^42^ Stress management has also been shown to improve glycemic control in patients with Type 2 diabetes mellitus.^43^ The use of connected digital technology coupled with machine learning and AI could make huge strides in informing the underlying stress-related origins of disease activity in these chronic conditions and others, such as cancer, migraines, and arthritis. Using this new information could provide researchers with the tools to enable individuals and healthcare providers to detect, act, and intervene when acute stressors occur in real time.

Other exciting opportunities lie in disease prevention in high-risk populations or those entered periods of biological or social change. Adolescence and emerging adulthood is the peak onset time for psychiatric conditions and suicide-related behavior, and is a particularly risky time among adolescents with a positive psychiatric family history.^44^ The ability to monitor acute stressors in real time and learn individual reactions to them could unlock huge opportunity for prevention of disease during high-stress periods in life, such as the transition into university or physiological transitions such as pregnancy or even menopause. Imagine the scientific utility of a multidimensional map of interconnected biological signs of stress during pregnancy in understanding the early signs of impending obstetric complications such as preeclampsia and the potential tools this could offer women and their clinicians to monitor and detect early warning signs of these complications. Periods of reproductive transitions such as pregnancy or menopause reflect optimal-learning settings to explore the feasibility of detecting multidimensional stress responses given the natural adjustments in physiological responses during this time and the direct effect that stress exerts on these normally occurring processes. Other examples include high-stress work environments. Nurses or midwives are in need for support in an increasingly demanding workforce landscape with high rates of burnout.^45^ This high-stress environment provides another unique opportunity to test feasibility of these approaches, while simultaneously testing potential digital interventions to support stress management. Although, the potential for employers to take advantage of this information to monitor employee performance must be considered and addressed through rigorous data protection and privacy guidelines.

## Challenges

The promise of applying digital devices to health and wellness is recognized, although in parallel to several cautions of their use.^46,47^ Online surveys suggest those more likely to engage with wearable devices for health-monitoring purposes are younger and from higher socioeconomic backgrounds,^48^ although characteristics of digital tool users versus non-users are largely unknown. This poses challenges from a feasibility and ethical standpoint in terms of equitable access and reaching vulnerable populations that will benefit most from these tools.^49^ The information produced from integrated smartphone apps and wearable devices is at the heart of big data, involving high volume, variety, velocity, and variability of data across different venues coinciding with further feasibility and ethical challenges. The scale of data produced from integrated digital tools will require rigorous approaches towards data privacy, security, and sophisticated ETL pipelines.

The opportunity connected digital technology could offer for the return of human agency is promising but not without risks. The potential for negative or harmful reactions towards the returning of symptoms, particularly objective symptoms, or the monitoring of symptoms by clinicians is largely unknown. Further, connected digital devices aimed at detecting, tracking, and reducing stress may inadvertently induce stress through disruptions in daily life, sleep, and in real-world social interactions and relationships, through social comparisons, fear of missing out and by increasing daily hassles surrounding connectivity, battery life, and device malfunction. In parallel to pilot testing of these approaches, these potential risks should be explored.

Finally, stress is a normal and necessary human response. Defining the line where stress is adaptive versus harmful via connected digital devices will require careful thought and consideration so as to avoid another means to de-stress individuals from everything conflictual or disruptive, that might in actuality be necessary for resilience. It will be particularly important to consider this potential devaluing of stress for downstream implications of digital devices targeting young or vulnerable populations.

## Conclusions

The advancement of digital technologies involving wearable devices, smartphone phone apps, and machine learning and AI affords a unique opportunity to accelerate the field of stress research and leverage this technology for evidence-based care. These complementary approaches could provide the tools to identify a multidimensional longitudinal measure of the stress response in a naturalistic setting and unlock complex unknown patterns of the stress response in forecasting downstream consequences. In the short term, this potential of identifying digital biomarkers of impending chronic conditions could be a huge utility to the scientific and medical community for understanding the stress-related origins of disease through individual trajectories and developing and testing interventions from this knowledge. In the long-term, these innovative approaches could have the ability to bridge the gap between symptom change and healthcare visit by enabling and empowering individuals to monitor their stress outside a hospital setting and facilitate early detection by providing healthcare workers with continuous information to identify early warning signs. The implications this has for the return of agency and individualized care is extensive reflecting an exciting period with far-reaching opportunities.
